# Jump exercise during hindlimb unloading protect against the deterioration of trabecular bone microarchitecture in growing young rats

**DOI:** 10.1186/2193-1801-2-35

**Published:** 2013-01-31

**Authors:** Yong-In Ju, Teruki Sone, Kazuhiro Ohnaru, Hak-Jin Choi, Kyung-A Choi, Masao Fukunaga

**Affiliations:** 1 Department of Health and Sports Sciences, Kawasaki University of Medical Welfare, 288 Matsushima, Kurashiki, Okayama, 701-0193 Japan; 2 Department of Nuclear Medicine, Kawasaki Medical School, Kurashiki, Okayama, 701-0192 Japan; 3 Graduate School of Medical Professions, Kawasaki University of Medical Welfare, Kurashiki, Okayama, 701-0193 Japan; 4 Graduate School of Sport Science, Sungkyunkwan University, Suwon, Gyeonggi-Do, 440-746 Korea; 5 Kawasaki Medical School, Kurashiki, Okayama, 701-0192 Japan

**Keywords:** Jump exercise, Tail suspension, Trabecular bone, Microarchitecture, Microcomputed tomography

## Abstract

Three-dimensional femoral trabecular architecture was investigated in tail-suspended young growing rats and the effects of jump exercise during the period of tail-suspension were also examined. Eight-week-old male Wistar rats (n = 24) were randomly assigned to three body weight-matched groups: a tail suspended group (SUS, n = 8); a sedentary control group (CON, n = 8) and rats primed with jump exercise during the period of tail suspension (JUM, n = 8). The jump exercise protocol consisted of 30 jumps/day, five days/week with a 40 cm jump height. After 3 weeks of jump exercise, bone mineral density (BMD) of the entire right femur was measured using dual energy X-ray absorptiometry. Three-dimensional trabecular bone architecture at the distal femoral metaphysis was evaluated using microcomputed tomography (micro-CT). Tail suspension caused a decrease in femoral BMD (−5%, p < 0.001) and trabecular bone architectural deterioration. Deterioration in the trabecular network during hindlimb unloading was mostly attributed to the reduction of trabecular number (−32%, p < 0.001) in the distal femoral metaphysis. Jump exercise during the tail suspension period increased trabecular thickness (14%, p < 0.001) and the reduction of trabecular number was suppressed. The present data indicate that jump exercise applied during hindlimb unloading could be able to inhibit bone loss and trabecular bone architectural deterioration caused by tail suspension.

## Introduction

Long-duration exposure to a microgravity environment has been shown to have detrimental effects on the human skeletal system. Soviet/Russian Mir spacecraft and International Space Station data indicated that areal bone mineral density (aBMD) was lost at an average monthly rate of 1.06% at the spine and 1.0 - 1.6% at the hip (LeBlanc et al. 2000). Other studies have shown similar deleterious effects on bone during prolonged bed rest (Armbrecht et al. 2011) and after spinal cord injury (Modlesky et al. 2004).

The general consensus among exercise physiologists has been that exercise is needed as a countermeasure to maintain bone health in crews during and after spaceflight as well as in patients with disuse osteoporosis (Rittweger et al. 2005; Shackelford et al. 2004). In addition to exercise, various other techniques such as electrical stimulation, load suits, pharmacologic therapy, and artificial gravity have been considered to alleviate the negative effects of microgravity on the skeletal system (Rubin et al. 2002). Aerobic exercise has been used as a countermeasure strategy for crews in spaceflight since the days of Skylab. However, this countermeasure has done little to prevent the bone loss associated with a spaceflight (LeBlanc et al. 2000). Jump exercise seems to provide a promotion of bone formation superior to that of aerobic exercise such as running in both humans and animals (Judex and Zernicke 2000; Umemura et al. 1997). It has been well established that peak loads and loading rates are more important factors than the number of repetitions in maintaining bone mineral density (Rubin and Lanyon 1985). Therefore, jump exercise may provide more protection against disuse bone loss than aerobic exercise.

The rat hindlimb-elevation tail suspension model has been widely used to simulate the bone changes occurring with disuse, mimicking results from human disuse. We have previously reported that suspension-induced trabecular deterioration persists after remobilization and that jump exercise during the remobilization period could restore the integrity of trabecular architecture in the femur of young growing rats (Ju et al. 2008). To date, no published animal studies have evaluated the effects of jump exercise on maintaining bone architecture during periods of tail suspension. Previous investigations have sought to define the osteogenic effect of mechanical loading during periods of disuse. Rubin et al. (2001), employed low-magnitude high frequency mechanical signals, finding that 10 min/day of mechanical stimulation was effective in maintaining metaphyseal bone volume and did attenuate bone formation losses at the proximal tibia. Swift et al. (2011) recently demonstrated that high-intensity simulated resistance training completed every other day during a period of disuse can prevent the loss of cancellous bone mass and material properties. However, their results could differ from that of the physiological (exercise) model in trabecular bone architecture because these studies used artificial mechanical loading models under anesthesia. We have identified only one other study that has evaluated the effects of physical exercise (resistance training) on the protection against both muscular atrophy and BMD loss during hindlimb suspension (Fluckey et al. 2002). However, the three-dimensional (3D) details of the heterogeneous trabecular structures were not clarified in that study. The aim of the current study was to investigate the effectiveness of jump exercise on preserving bone mass and architectural deterioration of trabecular bone during hindlimb unloading.

## Methods

### Animal care

Seven week-old male Wistar rats (n = 24) were acquired by Clea Japan (Osaka, Japan) and individually housed in 35 × 35 × 35 cm metal-grill cages under standard laboratory conditions at a room temperature of 22 ± 1°C and a humidity of 60 ± 5% with lights on at 08:00 and off at 20:00. Rats were fed standard laboratory animal chow (MF; Oriental Yeast, Chiba, Japan) containing 1.15% calcium and 0.88% phosphorus and water *ad libitum*. The experimental protocol and all animal care described in this investigation were performed in compliance with the Kawasaki University of Medical Welfare Laboratory Animal Care and Use Committee rules (08–013). After one week of acclimation to the diet and new environment, rats were randomly assigned into three groups (n = 8 each): tail-suspended group (SUS); sedentary control group reared in the breeding cage (CON); and rats primed with jump exercise during the period of tail suspension (JUM). Body weight was measured three times/week. At the end of the experiment, rats were anesthetized with intraperitoneal pentobarbital sodium (0.1 ml/100 g of body weight) and killed by exsanguination from the abdominal aorta. After death, the right hindlimb muscles (soleus and gastrocnemius) of each rat were collected and immediately weighed. The right femur was excised from each rat and cleaned soft tissue. Femoral length was measured using a digital caliper. The femur was stored at −40°C until required for further measurements.

### Tail suspension

Hindlimb unloading by tail suspension was performed according to previously described procedures (Ju et al. 2012; Ju et al. 2008). Briefly, the tails were cleaned with 70% alcohol, removing all dead or dirty skin and dried absolutely. Rats were not anesthetized. Traction tape was loosely wrapped around the tail in a helical pattern starting at 1 cm from the base of the tail. A strip of traction tape 1 cm wide was preattached to a metal connector, attached to two-thirds the length of the tail along the lateral sides, and then secured by two strips of filament tape. One strip of filament tape was placed around the tail at the end of the body side of the traction tape, and a second strip was added about half-way up the tape. The filament tape was loosely applied to allow normal blood circulation, but tight enough so that the traction tape would not peel from the tail. The metal connector was connected by a wire to a swivel mounted at the top of the cage, allowing 360° free rotation. The rat forelimbs maintained contact with the cage floor, allowing the rats to move and freely access food and water. Rat tail ends were maintained at an angle of about 30° from the cage floor, and thus the back feet of the rat did not touch the grid floor. During the tail-suspension period, rats were carefully monitored several times a day to prevent restriction of tail growth and circulation and ensure adequate food and water intake, grooming behavior, urination and defecation. The tape was also replaced every three days in order to avoid damage to the tail. Control rats were allowed to move unconstrained around the cages, and were fed the average amount eaten by the suspended rats daily.

### Exercise protocol

Jumping exercise protocol details have previously been described (Ju et al. 2012; Ju et al. 2008). Rats in the jump exercise groups were individually placed at the bottom of a special wooden box surrounded with boards after removal from the tail suspension apparatus. The rats were initially forced with electrical stimulus to jump and grasp the top of the board with the forelimbs and climb up the board. The rat was then returned to the floor of the cage to repeat the procedure. As rats became accustomed to the jump exercise, the electrical stimulus was applied after a few exercise days. The jump exercise program consisted of 30 jumps/day, five days/week for three weeks. The initial height of the box was 15 cm, and this was gradually increased to 40 cm during the first week. The thirty jump protocol required approximately one minute to complete. Each jump exercise session was performed during the dark period at the same time each day. Rats in the SUS group were removed from the tail suspension state (for one min) while the JUM group rats were exercising. In order to avoid prolonged gravity environment, the JUM and SUS group rats were immediately returned to the tail suspension state at the end of jump exercise session.

### Bone mineral density (BMD) measurements

Femoral BMD was measured with dual energy X-ray absorptiometry using a QDR-2000 unit (Hologic, Waltham, MA, USA) at small animal ultra-high resolution scan mode, starting from the distal end of the femur, as previously reported (Joo et al. 2003). Briefly, the instrument was set on ultra-high resolution mode with line spacing of 0.254 mm, point resolution of 0.127 mm, and a collimator diameter of 0.9 mm. Excised femurs were placed in the supine position on the table and scanned while immersed in saline.

### Micro-CT scanning

**Figure 1:**
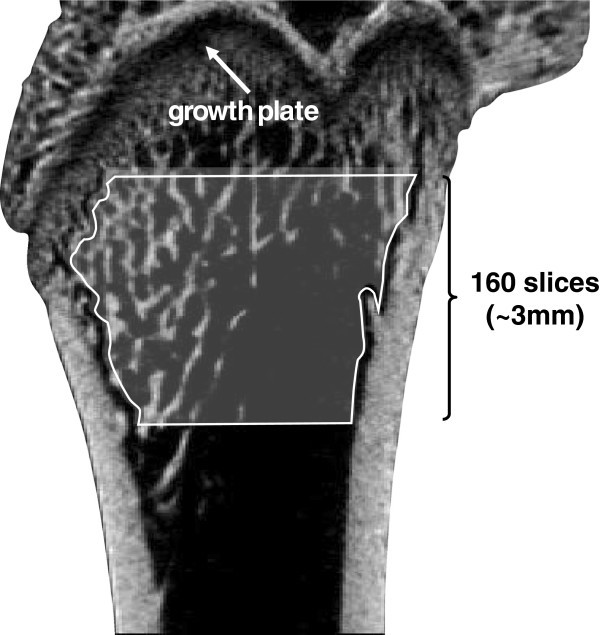
**Volumes of interest in the femur (sagittal section of distal femoral metaphysis).**

Three-dimensional trabecular microarchitecture of the right femur was evaluated using a micro-CT system (Ele Scan mini; Nittetsu Elex, Tokyo, Japan). This apparatus is based on a fan-beam tomography and is able to function in multislice mode. An X-ray tube with a microfocus (spot size of 6 × 8 μm) was which was able to attain a maximum resolution of 4 μm (in pixel size) was used. Study parameters included a source energy of 30 kVp and 70 mA to obtain the best contrast between bone and soft tissue. The sample area selected for scanning was positioned at a distance of 3–3.5 mm proximal from the distal femoral end, including the border between the distal metaphysis and growth plate. The distal femur was selected over the proximal femur because of the larger volume of cancellous bone available for 3D analysis in the distal femur. A total of 400 consecutive tomographic slices with a slice thickness of 19 μm (approximately 7.6 mm) were acquired. Digital data were reconstructed to obtain CT images with a pixel size of 21.9 μm in 512 × 512 matrices. After micro-CT scanning, the original image data were transferred to a workstation, and structural indices were calculated using 3D image analysis software (TRI/3D-BON; Ratoc System Engineering, Tokyo, Japan). The volume of interest was defined as the 160 slices above the most proximal portion of the growth plate (Figure 1). The resulting gray-scale images were segmented using a 3 × 3 median filter to remove noise, and a fixed threshold of 120 (0–255 range) to extract the mineralized bone phase. Isolated small particles in the marrow space and isolated small holes in bone were removed using a cluster-labeling algorithm. Cortical and trabecular bone were subsequently separated and structural indices calculated. Trabecular bone volume fraction (BV/TV), trabecular thickness (Tb.Th), trabecular number (Tb.N), and trabecular separation (Tb.Sp) were calculated by measuring 3D distances directly in the trabecular network (Hildebrand and Rüegsegger 1997).

### Data analysis

All data were expressed as means ± SD. All statistical analyses were carried out with the IBM SPSS Statistics 19.0 software package (IBM, Armonk, New York, USA). Comparisons among categories were statistically processed by one-way analysis of variance (ANOVA) followed by Tukey’s post hoc analysis. The level of statistical significance was set at p < 0.05.

## Results

### Body and hindlimb muscle weights and femoral length

Rat body weight, hindlimb muscle (soleus and gastrocnemius) weight, and femoral length from each group are shown in Table 1. At the end of the entire experimental period, tail suspended rats gained less body weight compared to the sedentary control group (12% and 10% difference in SUS and JUM, respectively; both p < 0.01). The weights of the soleus and gastrocnemius muscles in SUS group atrophied to 47% and 18% of those of the CON group, respectively. Atrophy of hindlimb muscle was not attenuated by jump exercise (39% in the soleus and 13% in the gastrocnemius muscle). Neither hindlimb unloading nor jump exercise affected femoral length.

**Table 1:** **Body weight, hindlimb muscle weight, and femoral length in experimental rats**

	CON	SUS	JUM
	(n = 8)	(n = 8)	(n = 8)
Initial body weight (g)	238.95 ± 7.55	242.11 ± 6.85	238.15 ± 19.44
Final body weight (g)	312.04 ± 15.50	274.61 ± 18.45**	279.43 ± 19.44**
Hindlimb muscles weight			
Soleus muscle (g)	0.12 ± 0.01	0.06 ± 0.01***	0.07 ± 0.01***
Gastrocnemius muscle (g)	1.40 ± 0.10	1.14 ± 0.08***	1.22 ± 0.11***
Total muscle mass (g)	1.52 ± 0.11	1.21 ± 0.09***	1.29 ± 0.11***
Femoral length (mm)	36.37 ± 0.87	36.32 ± 0.32	36.17 ± 0.53

All values represent mean ± SD. n, number of rats in each group; *SUS*, tail-suspended group; *CON*, sedentary cage control group for SUS; *JUM*, jump exercised group during tail suspension. Significant difference vs. CON group: **p < 0.01; ***p < 0.001.

### BMD of the femur

**Figure 2:**
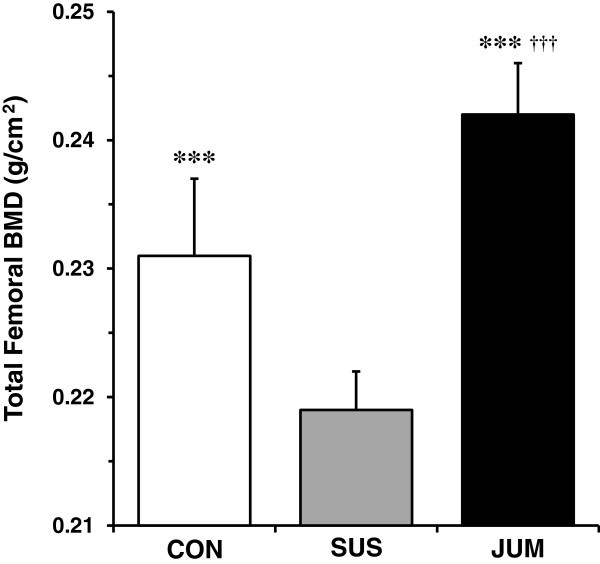
**Total femoral BMD measured by DXA.** All values represent mean ± SD. SUS, tail-suspended group; CON, sedentary control group for SUS; JUM, jump exercised group during tail suspension. Significant difference vs. SUS group: ^***^p < 0.001. Significant difference vs. CON group: ^†††^*p* < 0.001.

After three weeks of tail suspension, total femoral BMD in the SUS group showed 5% lower values when compared with the sedentary control rats (p < 0.001) (Figure 2). In contrast, jump exercise during the hindlimb unloading period resulted in significantly higher values of total femoral BMD compared with not only the SUS group (+ 10%, p < 0.001) but also the CON group (+ 5%, p < 0.001).

### Microstructural properties

**Figure 3:**
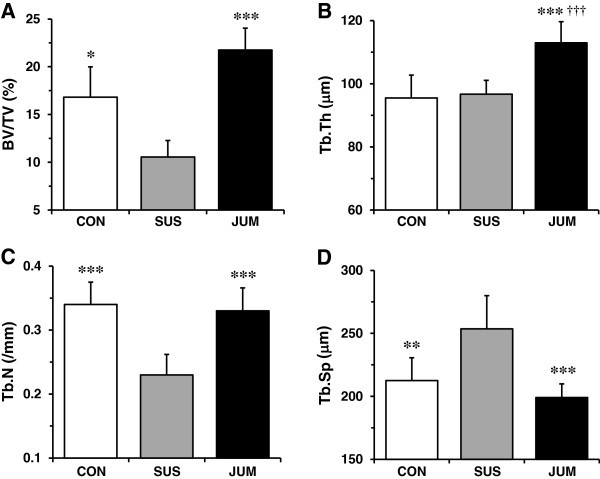
**Microstructural parameters in the distal femoral metaphysis measured by micro-CT.** All values represent mean ± SD. SUS, tail-suspended group; CON, sedentary control group for SUS; JUM, jump exercised group during tail suspension; BV/TV, trabecular bone volume (**A**); Tb.Th, trabecular thickness (**B**); Tb.N, trabecular number (**C**); Tb.Sp, trabecular separation (**D**). Significant difference vs. SUS group: ^*^p < 0.05; ^**^p < 0.01; ^***^p < 0.001. Significant difference vs. CON group: ^†††^*p* < 0.001.

**Figure 4:**
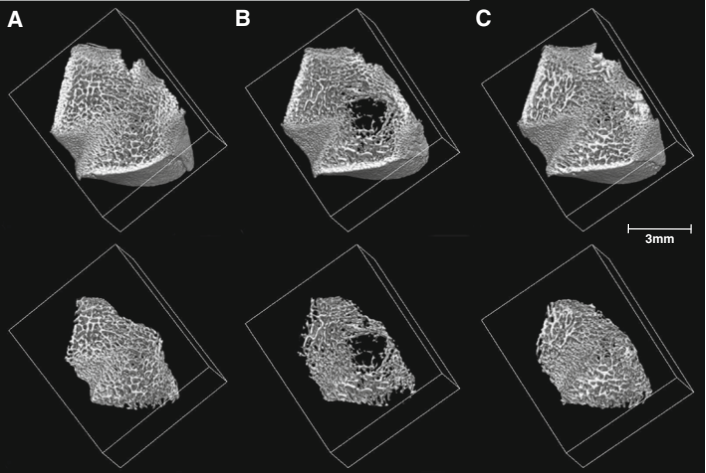
**Typical three-dimensional micro-computed tomography images of the distal femoral metaphysis: (A) CON; (B) SUS; and (C) JUM.** Intact bone and isolated cancellous bone for calculating trabecular bone parameters are shown at the top and bottom, respectively. SUS, tail-suspended group; CON, sedentary control group for SUS; JUM, jump exercised group during tail suspension.

Tail suspension induced a marked deterioration of trabecular architecture in the distal metaphysis of the femur (Figures 3 and 4). Compared with sedentary control rats, hindlimb unloading rats showed a decrease in BV/TV by −37% (p < 0.05), Tb.N by −32% (p < 0.001), and an increase in Tb.Sp by +16% (p < 0.01). No significant difference in Tb.Th was observed between the SUS and CON group. Jump exercise employed during unloading resulted in the greatest preventive effects against osteopenia, with increased BV/TV (+52%, p < 0.001), increased Tb.Th (+14%, p < 0.001), and reduced Tb.Sp (−22%, p < 0.001). Furthermore, jump-exercised rats during hindlimb unloading produced greater Tb.Th (+15%, p < 0.001) than sedentary control rats. On the other hand, Tb.N was not significantly altered by jump exercise. Figure 4 shows typical features of 3D trabecular microstructure in the distal femoral metaphysis for a rat from each group. These images demonstrate that tail suspension elicited marked deterioration in trabecular architecture, and that these changes recovered with jump exercise.

## Discussion

The major finding of the present study is that jump exercise during skeletal unloading showed a positive effect on the suppression of tail suspension-induced osteopenia at the femur in growing rats. Jump exercised rats retained cancellous bone mass in the distal femur, as evidenced by the increase in trabecular thickness, but did not have a change in trabecular number. These results suggest that jump exercise applied during hindlimb unloading can prevent the microarchitectural deterioration of trabecular bone by an increase in trabecular thickness while controlling the reduction of trabecular number in the distal femoral metaphysis.

Exercise has been widely accepted as being beneficial to skeletal bone. Various types of mechanical loading in rat models including treadmill running (Iwamoto et al. 1999), jumping (Umemura et al. 1997), whole body vibration (Tezval et al. 2011), swimming (Hart et al. 2001), and resistance exercise (Notomi et al. 2000) have been examined for any influence on bone loss and each has proved effective. However, the required time for daily exercise is much longer in treadmill running, vibration, swimming, and resistance exercise (for over 60 minutes) when compared to jump exercise (about one minute for 30 jumps). The effects of hindlimb unloading can be reversed after the rat is removed from the tail suspended state for an extended time. Such an exercise program requiring an extended length of time is unsuitable as a protocol during the hindlimb unloading state. Conversely, jump exercise has an advantage as an exercise program during the hindlimb unloading state, because it can be completed within approximately one minute. So far little is known about the effect of an exercise intervention during an unloading period on trabecular bone architecture. To the best of our knowledge, this study is the first to assess the effect of jump exercise on trabecular bone architecture during tail suspension.

A number of *in vivo* and *in vitro* studies have been conducted during spaceflight to clarify the mechanisms underlying the bone loss induced in a microgravity environment. However, the availability of spaceflight experiments is extremely limited. Ground-based studies have thus been conducted to investigate the mechanisms of osteopenia induced by weightlessness. The rat tail-suspension model has been well justified in previous studies as an appropriate model for the study of simulated bone changes induced by weightlessness (Martin 1990; Wronski and Morey-Holton 1987). Previous studies have reported that tail suspended rats display reduced trabecular bone mass, as evidenced by decreases in trabecular number and increases in trabecular separation (Basso et al. 2005; Ju et al. 2012; Ju et al. 2008). The present findings are consistent with these reports. In the present study, as expected, tail suspended rats demonstrated significantly decreased trabecular bone volume (−37%, p < 0.05) as a result of decreasing trabecular number (−32%, p < 0.001) and increasing trabecular separation (+19%, p < 0.001), and essentially no change in trabecular thickness in the distal femoral metaphysis. The tail suspended rats exhibited a loss of trabeculae, particularly from the central zone of the femur with a loss of trabecular number, although the remaining trabeculae did not show significant differences in thickness compared with control group (Figure 4). These results imply that loss in cancellous bone during 21 days of hindlimb unloading is predominantly due to decreases in trabecular bone number as opposed to trabecular thickness. This assumption is supported by the work of Basso et al. (Basso et al. 2005), who demonstrated that deterioration in the trabecular network after 14 days of skeletal unloading are mostly attributable to decreases in trabecular number with slight thinning of trabecular thickness. This phenomenon has also been reported in the ovariectomized rat model (Chen et al. 2001; Ito et al. 2002). Furthermore, similar findings have been confirmed in humans subjected to unloading caused by spinal cord injury (Modlesky et al. 2004) and prolonged bed rest (Armbrecht et al. 2011).

Several previous histomorphometric analyses have found that the increase in trabecular bone mass that occurs with resistance exercise is primarily due to increased trabecular thickness, rather than noticeable changes in numbers of trabeculae (Holy and Zerath 2000; Notomi et al. 2000; Notomi et al. 2001). Moreover, we recently found that restoration of trabecular bone architecture induced by jump exercise during remobilization is predominantly attributable to increased trabecular thickness (Ju et al. 2012). The present findings are consistent with previous studies where jump exercise applied during a skeletal unloading period produced a significant increase in distal femur trabecular bone mass, primarily by increased trabecular thickness without significant change in trabecular number. These results imply that the cancellous bone gain induced by jump exercise during hindlimb unloading is predominantly attributable to increases in trabecular thickness while controlling the reduction in trabecular number. In contrast, Swift et al. (2011) employing simulated resistance training during periods of disuse, found that muscle contractions once every three days produced significantly larger increases in proximal tibial cancellous bone mass associated both with greater trabecular thickness and number than untreated hindlimb unloaded rats. The reason for the apparent discrepancy between these studies is twofold. First, the difference in the mode of mechanical stimulation applied during hindlimb unloading might produce the difference in trabecular architecture. Since Swift et al. (2011) used the low-magnitude, high-frequency mechanical stimulation, the distribution and duration of mechanical stress in bone by the stimulation could be substantially different from the stress induced by jump exercise. In humans, high-impact activity such as jump exercise elicits greater bone gain than repetitive high-frequency activities such as running or walking (Fuchs et al. 2001; Kohrt et al. 2004). Thus, different mechanical loading activities may have different mechanisms of action on the trabecular structural characteristics. Further study is needed to elucidate the mechanism of differential effects among different types of mechanical loading on trabecular bone architecture. It is also possible that different findings are related to differing assessment methods (i.e., analyses of the 3D trabecular bone microarchitecture by micro-CT versus 2D trabecular bone microarchitecture by histomorphometry). The volume of interest provided by 2D sections may not accurately reflect the change across the entire volume of the metaphyseal region. However, the micro-CT may allow for the assessment of true 3D quantification of trabecular bone microarchitecture.

Changes in body weight and muscle mass may play important roles in the regulation of bone mass. Both body weight and hindlimb muscle mass have decreased in rats subjected to hindlimb unloading with tail suspension. A greater weight loss during hindlimb unloading was observed in slow-twitch soleus muscle (47%) than in the fast-twitch gastrocnemius muscle (18%), which is consistent with previous findings (Hurst and Fitts 2003). It has been well documented that the soleus muscle plays a major role in anti-gravity function and is highly dependent on gravity for the normal expression of protein mass and slow phenotype; whereas the gastrocnemius muscle is physically active and does not have the same type of anti-gravity function. During hindlimb unloading, anti-gravity muscles such as the soleus are therefore more vulnerable to atrophy than the gastrocnemius muscle. In the present study, hindlimb muscle atrophy was not fully prevented with jump exercise. Nevertheless, the final bone volume was greater in the JUM group than in the CON and SUS groups. A similar effect was described by Notomi et al. (2003), who reported that the climbing exercise increased bone mass and strength without significantly increasing hindlimb muscle weight after 8 weeks of exercise. In the jump exercise protocol that was used, the bones of the lower limb were loaded only by the ground-reaction force and muscle contraction force at the point of jump take-off. There was no ground-reaction force at landing because the rats were gently placed with their forelimbs on the floor by a technician. These data suggest that the suppression of bone loss and architectural deterioration observed in jump-exercised rats is derived primarily from the mechanical stress generated by the ground-reaction or muscle contraction occurring at take-off.

The present study displays several limitations. First, the study was conducted on the rat hindlimb unloading model combined with the jump exercise training. Experimental animals such as rats are useful for defining biological mechanisms but findings are not necessarily directly applicable to the human situation, since rat bone-modeling patterns are different from human patterns. Furthermore, jump exercise is actually difficult to apply to astronauts during missions or patients with disuse osteoporosis. In the present study, jump exercise was performed in a gravity environment after removal from the tail suspension apparatus. Our results might differ if the same exercise was performed in space. Hence, specially designed workout equipment has to be developed to provide the stimulus to bone, in order to apply this methodology to astronauts or patients with disuse osteoporosis. Nonetheless, the results of this study will improve our understanding of the architectural adaptation of trabecular bone with mechanical stimulation applied during skeletal unloading such as weightlessness and immobility. Bone loss and architectural deterioration caused by inactivity and immobility is one of the major factors for osteoporosis. Recently, several anabolic agents for the treatment of osteoporosis are being developed, which could partly share mechanisms of action with the exercise stimulation. Thus, our data may provide valuable insights into the development of therapeutic agents with effect similar to exercise stress. Secondly, we did not perform the histomorphometric analysis and cannot confirm tissue-level mechanisms for the functional adaptation of the trabecular architecture to mechanical loading observed in the present study. Further study will be needed to clarify the mechanism of mechanical stimulation on trabecular bone architecture.

## Conclusion

In conclusion, three weeks of simulated microgravity by tail suspension resulted in a reduction of cancellous bone mass in the distal femur due to changes in trabecular number. Jump exercise during unloading inhibited bone loss by increasing trabecular thickness, as well as by suppressing the loss of trabecular number. The findings from this study contribute to our understanding of skeletal adaptation by mechanical stimulation and may assist in developing novel exercise strategies for bone loss in disuse osteoporosis.

## References

[CR1_120] Armbrecht G Belavý DL Backström M Beller G Alexandre C Rizzoli R Felsenberg D Trabecular and cortical bone density and architecture in women after 60 days of bed rest using high-resolution pQCT: WISE 2005 J Bone Miner Res 2011 26 2399 3410 10.1002/jbmr.482 21812030

[CR2_120] Basso N Jia Y Bellows CG Heersche JN The effect of reloading on bone volume, osteoblast number, and osteoprogenitor characteristics: studies in hindlimb unloaded rats Bone 2005 37 370 378 10.1016/j.bone.2005.04.033 16005699

[CR3_120] Chen JL Yao W Frost HM Li CY Setterberg RB Jee WS Bipedal stance exercise enhances antiresorption effects of estrogen and counteracts its inhibitory effect on bone formation in sham and ovariectomized rats Bone 2001 29 126 133 10.1016/S8756-3282(01)00496-3 11502473

[CR4_120] Fluckey JD Dupont-Versteegden EE Montague DC Knox M Tesch P Peterson CA Gaddy-Kurten D A rat resistance exercise regimen attenuates losses of musculoskeletal mass during hindlimb suspension Acta Physiol Scand 2002 176 293 300 10.1046/j.1365-201X.2002.01040.x 12444935

[CR5_120] Fuchs RK Bauer JJ Snow CM Jumping improves hip and lumbar spine bone mass in prepubescent children: a randomized controlled trial J Bone Miner Res 2001 16 148 156 10.1359/jbmr.2001.16.1.148 11149479

[CR6_120] Hart KJ Shaw JM Vajda E Hegsted M Miller SC Swim-trained rats have greater bone mass, density, strength, and dynamics J Appl Physiol 2001 91 1663 1668 11568148 10.1152/jappl.2001.91.4.1663

[CR7_120] Hildebrand T Rüegsegger P A new method for the model-independent assessment of thickness in three-dimensional images J Microscopy 1997 185 67 75 10.1046/j.1365-2818.1997.1340694.x

[CR8_120] Holy X Zerath E Bone mass increases in less than 4 wk of voluntary exercising in growing rats Med Sci Sports Exerc 2000 32 1562 1569 10994905 10.1097/00005768-200009000-00006

[CR9_120] Hurst JE Fitts RH Hindlimb unloading-induced muscle atrophy and loss of function: protective effect of isometric exercise J Appl Physiol 2003 95 1405 1417 12819219 10.1152/japplphysiol.00516.2002

[CR10_120] Ito M Nishida A Nakamura T Uetani M Hayashi K Differences of three-dimensional trabecular microstructure in osteopenic rat models caused by ovariectomy and neurectomy Bone 2002 30 594 598 10.1016/S8756-3282(02)00684-1 11934651

[CR11_120] Iwamoto J Yeh JK Aloia JF Differential effect of treadmill exercise on three cancellous bone sites in the young growing rat Bone 1999 24 163 169 10.1016/S8756-3282(98)00189-6 10071907

[CR12_120] Joo YI Sone T Fukunaga M Lim SG Onodera S Effects of endurance exercise on three-dimensional trabecular bone microarchitecture in young growing rats Bone 2003 33 485 493 10.1016/S8756-3282(03)00212-6 14555251

[CR13_120] Ju YI Sone T Ohnaru K Choi HJ Fukunaga M Differential effects of jump versus running exercise on trabecular architecture during remobilization after suspension-induced osteopenia in growing rats J Appl Physiol 2012 112 766 772 10.1152/japplphysiol.01219.2011 22162526

[CR14_120] Ju YI Sone T Okamoto T Fukunaga M Jump exercise during remobilization restores integrity of the trabecular architecture after tail suspension in young rats J Apple Physiol 2008 104 1594 1600 10.1152/japplphysiol.01004.2007 18420719

[CR15_120] Judex S Zernicke RF High-impact exercise and growing bone: relation between high strain rates and enhanced bone formation J Appl Physiol 2000 88 2183 2191 10846034 10.1152/jappl.2000.88.6.2183

[CR16_120] Kohrt WM Bloomfield SA Little KD Nelson ME Yingling VR American college of sports medicine. American college of sports medicine position stand: physical activity and bone health Med Sci Sports Exerc 2004 36 1985 1996 10.1249/01.MSS.0000142662.21767.58 15514517

[CR17_120] LeBlanc A Schneider V Shackelford L West S Oganov V Bakulin A Voronin L Bone mineral and lean tissue loss after long duration space flight J Musculoskelet Neuronal Interact 2000 1 157 160 15758512

[CR18_120] Martin RB Effects of simulated weightlessness on bone properties in rats J Biomech 1990 23 1021 1029 10.1016/0021-9290(90)90317-V 2229085

[CR19_120] Modlesky CM Majumdar S Narasimhan A Dudley GA Trabecular bone microarchitecture is deteriorated in men with spinal cord injury J Bone Miner Res 2004 19 48 55 10.1359/jbmr.0301208 14753736

[CR20_120] Notomi T Lee SJ Okimoto N Okazaki Y Takamoto T Nakamura T Suzuki M Effects of resistance exercise training on mass, strength, and turnover of bone in growing rats Eur J Appl Physiol 2000 82 268 274 10.1007/s004210000195 10958368

[CR21_120] Notomi T Okimoto N Okazaki Y Tanaka Y Nakamura T Suzuki M Effects of tower climbing exercise on bone mass, strength, and turnover in growing rats J Bone Miner Res 2001 16 166 174 10.1359/jbmr.2001.16.1.166 11149481

[CR22_120] Notomi T Okimoto N Okazaki Y Nakamura T Suzuki M Tower climbing exercise started 3 months after ovariectomy recovers bone strength of femur and lumbar vertebrae in aged osteopenic rats J Bone Miner Res 2003 18 140 149 10.1359/jbmr.2003.18.1.140 12510816

[CR23_120] Rittweger J Frost HM Schiessl H Ohshima H Alkner B Tesch P Felsenberg D Muscle atrophy and bone loss after 90 days’ bed rest and the effects of flywheel resistive exercise and pamidronate: results from the LTBR study Bone 2005 36 1019 1029 10.1016/j.bone.2004.11.014 15811637

[CR24_120] Rubin C Turner AS Mallinckrodt C Jerome C McLeod K Bain S Mechanical strain, induced noninvasively in the high-frequency domain, is anabolic to cancellous bone, but not cortical bone Bone 2002 30 445 452 10.1016/S8756-3282(01)00689-5 11882457

[CR25_120] Rubin C Xu G Judex S The anabolic activity of bone tissue, suppressed by disuse, is normalized by brief exposure to extremely low-magnitude mechanical stimuli FASEB J 2001 15 2225 2229 10.1096/fj.01-0166com 11641249

[CR26_120] Rubin CT Lanyon LE Regulation of bone mass by mechanical strain magnitude Calcif Tissue Int 1985 37 411 417 10.1007/BF02553711 3930039

[CR27_120] Shackelford LC LeBlanc AD Driscoll TB Evans HJ Rianon NJ Smith SM Spector E Feeback DL Lai D Resistance exercise as a countermeasure to disuse-induced bone loss J Appl Physiol 2004 97 119 129 10.1152/japplphysiol.00741.2003 15220316

[CR28_120] Swift JM Swift SN Nilsson MI Hogan HA Bouse SD Bloomfield SA Cancellous bone formation response to simulated resistance training during disuse is blunted by concurrent alendronate treatment J Bone Miner Res 2011 26 2140 2150 10.1002/jbmr.407 21509821

[CR29_120] Tezval M Biblis M Sehmisch S Schmelz U Kolios L Rack T Stuermer KM Stuermer EK Improvement of femoral bone quality after low-magnitude, high-frequency mechanical stimulation in the ovariectomized rat as an osteopenia model Calcif Tissue Int 2011 88 33 40 10.1007/s00223-010-9423-7 21052653 PMC3021189

[CR30_120] Umemura Y Ishiko T Yamauchi T Kurono M Mashiko S Five jumps per day increase bone mass and breaking force in rats J Bone Miner Res 1997 12 1480 1485 10.1359/jbmr.1997.12.9.1480 9286765

[CR31_120] Wronski TJ Morey-Holton ER Skeletal response to simulated weightlessness: a comparison of suspension techniques Aviat Space Environ Med 1987 58 63 68 3814035

